# Long-Term Assessment of Contemporary Ion-Releasing Restorative Dental Materials

**DOI:** 10.3390/ma15124042

**Published:** 2022-06-07

**Authors:** Danijela Marovic, Matej Par, Karlo Posavec, Ivana Marić, Dominik Štajdohar, Alen Muradbegović, Tobias T. Tauböck, Thomas Attin, Zrinka Tarle

**Affiliations:** 1 Department of Endodontics and Restorative Dentistry, School of Dental Medicine, University of Zagreb, Gunduliceva 5, 10000 Zagreb, Croatia; marovic@sfzg.hr (D.M.); dstajdohar@sfzg.hr (D.Š.); tarle@sfzg.hr (Z.T.); 2 Private Dental Practice, Dr. Ivana Novaka 28, 40000 Čakovec, Croatia; karlo.posavec.ned@gmail.com; 3 Private Dental Practice, Odranska 10, 10000 Zagreb, Croatia; ivana.maric912@gmail.com; 4 Private Dental Practice, Malkočeva 3, 75000 Tuzla, Bosnia and Herzegovina; muradbegovic.alen@gmail.com; 5 Clinic of Conservative and Preventive Dentistry, Center for Dental Medicine, University of Zurich, Plattenstrasse 11, 8032 Zurich, Switzerland; tobias.tauboeck@zzm.uzh.ch (T.T.T.); thomas.attin@zzm.uzh.ch (T.A.)

**Keywords:** flexural strength, modulus, water sorption, solubility, degree of conversion, alkasite, giomer, glass-ionomer, long-term

## Abstract

The objective was to evaluate new commercially available ion-releasing restorative materials and compare them to established anti-cariogenic materials. Four materials were tested: alkasite Cention (Ivoclar Vivadent) in self-cure or light-cure mode, giomer Beautifil II (Shofu), conventional glass-ionomer Fuji IX (GC), and resin composite Tetric EvoCeram (Ivoclar Vivadent) as a control. Flexural strength, flexural modulus, and Weibull modulus were measured one day, three months, and after three months with accelerated aging in ethanol. Water sorption and solubility were evaluated for up to one year. Degree of conversion was measured during 120 min for self-cured and light-cured Cention. In this study, Beautifil II was the ion-releasing material with the highest flexural strength and modulus and with the best resistance to aging. Alkasite Cention showed superior mechanical properties to Fuji IX. Weibull analysis showed that the glass-ionomer had the least reliable distribution of mechanical properties with the highest water sorption. The solubility of self-cured alkasite exceeded the permissible values according to ISO 4049. Degree of conversion of light-cured Cention was higher than in self-cure mode. The use of alkasite Cention is recommended only in the light-cure mode.

## 1. Introduction

In recent years, restorative dentistry has gradually shifted from “biocompatibility” to “bioactivity”. With the advancement of minimally invasive dentistry, scientific interest in restorative materials with ion release also increases [1,2,3,4,5]. Restorative materials should possess anti-demineralizing and remineralizing properties to fight against caries while retaining their stability over time and resistance to occlusal load, thermal changes, and enzymatic influences in the oral cavity.

The term “bioactive dental restorative materials” is still a matter of debate. While some biomaterial scientists claim that a bioactive material should be able to form a hydroxyapatite precipitate at its surface [6], others discard this idea [7]. At the same time, they should create an active interface with biological tissue [8]. Glass-ionomers were the first dental restorative materials able to satisfy some of the properties desired for the bioactive restorative material [9,10]. Fluoride release is considered accountable for promoting biomineralization of mineral-depleted hydroxyapatite [6,11], while self-adhesion to tooth substrate enables their direct interaction with hard dental tissues [9]. Glass-ionomers are hydrophilic materials and need water for their setting reaction. Still, they are also sensitive to dehydration (leading to cracking of the material surface) [12] and excessive water uptake (leading to the dissolution of metal cations) [13]. The clinical applicability of glass-ionomers is limited to low-stress bearing areas because of poor flexural strength, toughness, and wear [14,15].

Aiming to improve the mechanical properties and durability of conventional glass-ionomers to the level of resin composites [16,17,18], a variety of ion-releasing materials based on fluoroaluminosilicate glass as a filler component has been made: cermets, fiber-reinforced glass-ionomers, resin-modified glass-ionomers, compomers, and giomers [14,19]. The coupling of functional fillers and the methacrylate matrix is desirable to allow quick and on-demand hardening of a material. Giomers typically contain a resin-based matrix and unique pre-reacted glass-ionomer (PRG) fillers, which have a conventional glass core with a surface glass-ionomer layer pretreated with polyalkenoate acid and a completed acid–base reaction. PRG fillers are afterward dehydrated and silanated to ensure copolymerization to the resin [10]. Besides PRG fillers, giomers contain conventional silanated macro- and micro-fillers. This approach seems to be highly successful in terms of the giomer’s high fracture toughness and flexural strength [19,20]. The fluoride release depends on the material’s water sorption after placement in the moist environment and is therefore significantly lower than in resin-modified glass-ionomers or compomers [10,19]. Their behavior is considered very similar to resin composite, and their clinical performance is satisfactory [21].

Recently, a new class of resin-based ion-releasing materials appeared on the market, named alkasite materials. The name is derived from their alkalizing properties due to the release of hydroxide (OH^−^) ions. The only material in that class is produced by Ivoclar Vivadent (Schaan, Liechtenstein), whose composition was modified over time. Cention N (Ivoclar Vivadent) was the first material that appeared on the market in the hand-mix version. Cention (Ivoclar Vivadent) and Cention forte (Ivoclar Vivadent) are their successors in a capsulated version. According to the manufacturer, the composition of Cention is the same as that of Cention forte, the difference being in the application mode (Cention forte is recommended for use with a special adhesive system). Three main types of fillers are present: silanized inert barium aluminum silicate glass, calcium barium aluminum fluorosilicate glass similar to glass-ionomers, and calcium fluorosilicate glass or “alkasite” glass. Besides these components, the manufacturer states that Cention also contains ytterbium trifluoride and a prepolymerized filler termed Isofiller, similar to other materials from the same manufacturer. The liquid phase consists of dimethacrylates without any acidic groups that would impart self-adhesive properties [10]. Cention is a bulk-fill restorative material with photoinitiators and chemical catalysts enabling a dual-cure polymerization mechanism. This material releases Ca^2+^, F^−^ and PO_4_^3−^ ions in neutral and acidic conditions, leading to apatite formation on its surface [22,23]. A series of studies by Par et al. showed that Cention has an acid-neutralizing capability [4] and prevented demineralization of enamel [24] and dentine [25] when subjected to lactic acid over a prolonged period. Presently, this material is considered the only true commercially available bioactive composite [10,23]. Clinical studies are still lacking, as well as the investigations on the influence of mineral deposits at the surface of the restoration on proper oral hygiene maintenance and the antimicrobial action [26,27].

The release of ions or any other substances from a restorative material always raises concerns about the possible dissolution of functional filler particles. In the set material placed in an aqueous environment, this could create voids and facilitate water sorption, propagating further dissolution. Internal porosities lower the resistance of restoration to occlusal forces and facilitate their fracture [28]. A compromise between satisfactory mechanical properties and the ion-releasing benefits is needed. While mechanical properties of resin composites [18,29,30], glass-ionomers [14,15], and giomers [19,21] are sufficiently explored, studies focused on alkasite materials are scarce and mainly investigate the powder-liquid hand-mixed Cention N [31,32,33,34]. Besides the work of Par and co-workers [4,22,24,25] that focused on ion-releasing properties of Cention, a PubMed search of articles including the capsulated version of Cention resulted in finding only three papers studying fluoride release [35], wear behavior [36], or biologic effects on pulp cells [37]. The data about the long-term mechanical behavior of capsulated alkasite Cention used in either self-cure or light-cure mode is still lacking, especially considering the compositional modifications of the capsulated version in contrast to the predecessor Cention N.

This study was thus conducted to examine the long-term influence of water and aging on the mechanical properties of currently available ion-releasing materials. Six parameters were tested: flexural strength and modulus, Weibull modulus, degree of conversion, water sorption, and solubility. The null-hypotheses were: (I) there is no difference between different materials in any of the tested parameters, (II) for any given parameter, there is no difference between different time points, and (III) there is no difference between Cention when light-cured or self-cured in any of the tested parameters.

## 2. Materials and Methods

Four materials were tested in this study (Table 1), but with five testing groups, as one material, alkasite Cention, was tested in a light-cured (LC) and self-cured (SC) mode.

### 2.1. Study Protocol

**Figure 1:**
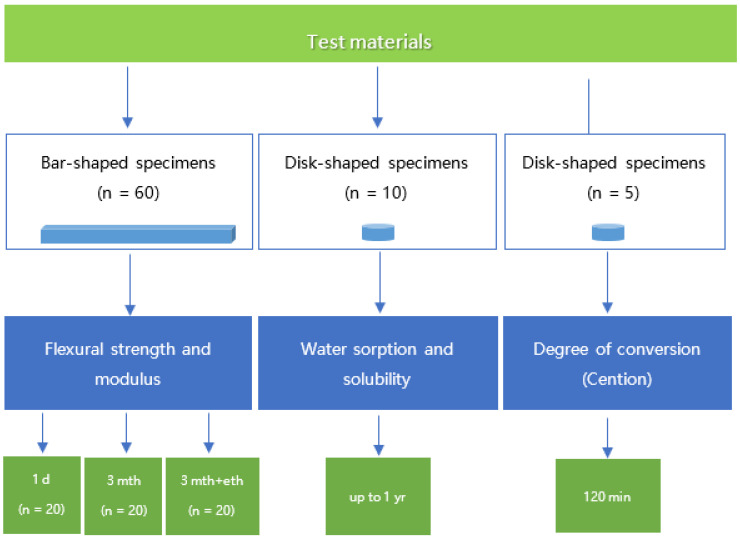
Flow chart of the study design.

Three tests were performed (degree of conversion, three-point bending, and water sorption), and six parameters were measured: flexural strength, flexural modulus, Weibull modulus, water sorption, solubility, and degree of conversion (only for Cention). The study design is depicted in Figure 1.

### 2.2. Three-Point Bending Test

For the three-point bending test, bar-shaped specimens with dimensions 16 × 2 × 2 mm were made [24]. Unset materials were filled in a custom-made silicone mold (Elite HD+Putty, Zhermack, Badia Polesine, Italy) in excess, pressed with a polyethylene terephthalate (PET) foil, and a microscope cover glass and flash material was removed. Light-curing was performed for Cention LC, Beautifil II, and Tetric EvoCeram using Bluephase G2 (Ivoclar Vivadent) with 950 mW/cm^2^ for 20 s, with three overlapping exposures on each side, making six irradiations in total. The radiant exitance of the curing unit was measured using a calibrated and NIST-referenced UV–Vis spectrophotometer (MARC; BlueLight Analytics, Halifax, NS, Canada) and amounted to 952 mW/cm^2^ with peak intensities at 405 and 457 nm. Cention SC and Fuji IX were left to set at room temperature for 15 min in the dark. All specimens were then immersed in distilled water and stored at 37 °C in the dark.

Sixty specimens per group were subjected to a three-point bending test using a customized universal testing machine (Ultratester, Ultradent Products Inc., South Jordan, UT, USA). Twenty specimens in each group were tested after one day in distilled water. Another 20 specimens were tested after three months (90 d) in distilled water, while the remaining 20 specimens were tested after storage in distilled water for three months, followed by immersion in absolute ethanol for three days. Flexural strength and modulus were calculated [25]. The Weibull analysis (reliability analysis) was performed by plotting the function ln:

ln (1/(1 − P_f_)) = m (ln σ − ln σ_θ_) (1)

where P_f_ = probability of failure, m = Weibull modulus σ = strength at failure, and σ_θ_ = characteristic strength.

### 2.3. Water Sorption and Solubility

Ten disk-shaped specimens per material were made (2 mm high and 6 mm in diameter) in Teflon molds. The setting of the materials was performed similarly to the three-point bending test: Cention LC, Beautifil II, and Tetric EvoCeram were light-cured with the identical curing unit for 20 s on each side. At the same time, Cention SC and Fuji IX were left to set at room temperature for 15 min in the dark.

After initial drying in the desiccator, the specimens were weighted with an analytical scale (NBL 254 i, Adam Equipment, Milton Keynes, UK). The obtained values were designated as the initial mass of the specimen (m_1_). Afterward, the specimens were individually placed in conical-shaped Eppendorf tubes with 4 mL of distilled water. They were stored for one year (365 days) at 37 °C in the dark. The mass of the specimens was weighted after 1, 7, 14, 90, 180, and 365 days (m_2_(t), t—time). After the immersion, the specimens were again dried in a desiccator. Their mass was regularly monitored until stable values (not differing from a previous measurement for more than 0.1 mg) were achieved. The final mass of the specimens after drying was marked as m_3_.

Water sorption and solubility were calculated according to the formula provided by ISO 4049 [25]:

water sorption = (m_2_(eq) − m_3_) (g) (2)

solubility = m_1_ − m_3_ (g) (3)

where m_2_(eq) represents mass equilibrium.

### 2.4. Degree of Conversion

The degree of conversion was measured for alkasite Cention in self-cure or light-cure mode, using Fourier transform infrared (FTIR) spectrometer (Nicolet iS50, Thermo Fisher, Madison, NJ, USA) with an attenuated total reflectance (ATR) accessory. Cention capsules were mixed, and the material was extruded directly on the diamond ATR crystal using custom-made silicone molds at room temperature (22 ± 1 °C). The specimens (d = 6 mm, h = 1.5 mm) were covered with PET strips and left to self-cure or light-cured for 20 s using Bluephase G2. The curing unit was positioned perpendicularly and in direct contact with the composite specimen surface. FTIR spectra were continuously collected at a rate of 2 spectra per second for 120 min after the placement of the material or start of light-activated curing, with 4 scans and a resolution of 8 cm^−1^ [26]. Five specimens per experimental group were tested (n = 5).

The ratio between the peak heights of aliphatic (1638 cm^−^^1^) and aromatic (1608 cm^−^^1^) bands were used to calculate the degree of conversion (DC) for each spectrum for uncured and cured specimens. The degree of conversion was plotted against time.

(4) $$\mathrm{DC}\;\left(\%\right)=\left(1\;-\;\frac{{(1638\;\mathrm{cm}}^{-1}/1608\;{\mathrm{cm}}^{-1})\;\mathrm{after}\;\mathrm{curing}}{{(1638\;\mathrm{cm}}^{-1}/1608\;{\mathrm{cm}}^{-1})\;\mathrm{before}\;\mathrm{curing}}\right)\;\times \;100\%$$

### 2.5. Statistical Analysis

The normality of distribution was evaluated using Shapiro Wilk’s test and the inspection of normal Q-Q diagrams. Since the data for flexural strength and modulus data violated the assumption of normality, the comparisons performed were statistically analyzed using the Kruskal-Wallis test with Bonferroni post-hoc adjustment. Weibull statistics were performed to examine the reliability of the materials. For water sorption and solubility, data were normally distributed, hence why the mixed-model ANOVA with Tukey and Bonferroni corrections (for independent and dependent observations, respectively) were used for statistical analysis. The degree of conversion data for Cention SC and Cention LC were normally distributed and compared using a *t*-test for independent observations. SPSS (version 20, IBM, Armonk, NY, USA) was used for the statistical analysis with the level of significance α = 0.05.

## 3. Results

**Figure 2:**
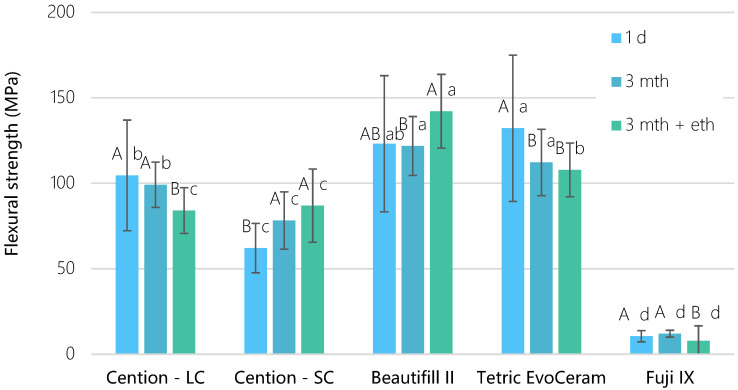
Flexural strength as a function of time for tested materials (mean values ± standard deviation, n = 20). Identical uppercase letters denote *p* > 0.05 for the same material between different time points; identical lowercase letters denote *p* > 0.05 between materials at the same time point.

Light-cured materials exhibited the highest flexural strength, followed by self-cured materials, in a decreasing manner: Tetric EvoCeram = Beautifil II > Cention LC > Cention SC > Fuji IX. Figure 2 shows that the flexural strength of the Cention LC was the highest after 1 day (104 ± 32 MPa) and after 3-month water exposure (99 ± 13 MPa), while significantly decreasing (*p* = 0.003) after ethanol immersion (84 ± 13 MPa). On the contrary, the same material showed a flexural strength increase when left to self-cure, so the 1-day values (62 ± 13 MPa) were significantly lower (*p* < 0.001) than values after 3-month water exposure (78 ± 16 MPa) and an additional ethanol immersion (87 ± 21 MPa, *p* < 0.05). Beautifil II demonstrated unexpectedly higher flexural strength values after 3-month water and ethanol exposure than after 3-month exposure to water only (*p* = 0.032).

**Figure 3:**
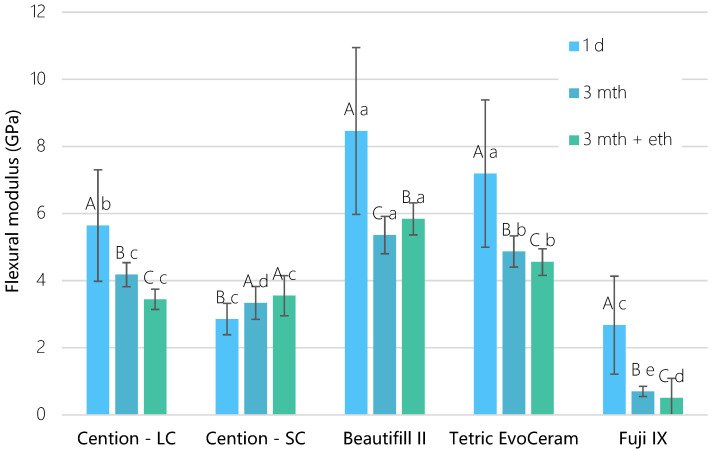
Flexural modulus as a function of time for tested materials (mean values ± standard deviation, n = 20). Identical uppercase letters denote *p* > 0.05 for the same material between different time points; identical lowercase letters denote *p* > 0.05 between materials at the same time point.

A similar pattern was noted for the flexural modulus, as depicted in Figure 3. Cention LC demonstrated a significant drop down (*p* < 0.001) in modulus after 3 mth (4.2 ± 0.3 GPa) and 3 mth + eth (3.4 ± 0.3 GPa) groups compared to 1-day values (5.6 ± 1.7 GPa). The flexural modulus of Cention SC was significantly lower (*p* = 0.001) after 1 day (2.8 ± 0.5 GPa) than after 3 mth (3.3 ± 0.5 GPa) and 3 mth + eth (3.5 ± 0.6 GPa). Beautifil II showed a higher modulus (*p* < 0.001) after ethanol exposure (5.8 ± 0.5 Gpa) than after 3-month water exposure (5.4 ± 0.6 GPa). Fuji IX had the significantly lowest (*p* < 0.001) flexural strength (7.9–12.0 MPa) and the lowest flexural modulus (0.5–2.7 GPa).

**Figure 4:**
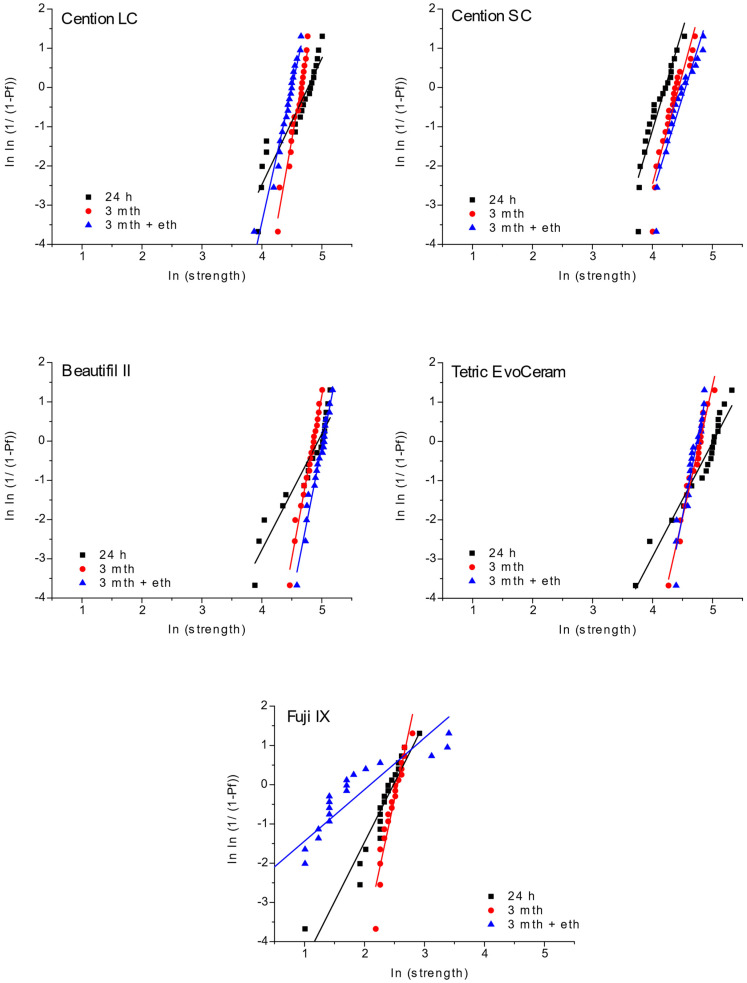
Weibull plots for tested materials and time points of measurement.

Material reliability was calculated by the Weibull analysis (Figure 4). All light-cured groups showed high reliability and similarly narrow distribution of values, except after one day of water immersion. Unlike them, Cention SC had higher reliability with closely distributed values for one day. The Cention SC group demonstrated similar values, but these values were slightly lower compared to Cention LC. Fuji IX showed a wide distribution of data and, therefore, much lower reliability than other materials in this study.

**Figure 5:**
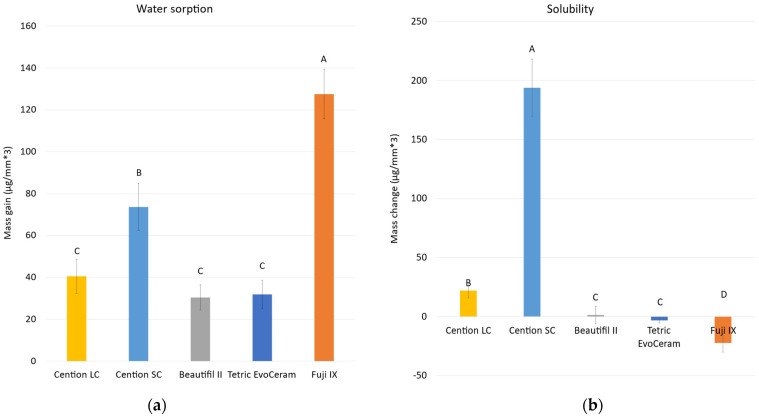
Water sorption (**a**) and solubility (**b**) for tested materials (mean values ± standard deviation, n = 10). Identical uppercase letters denote *p* > 0.05.

**Figure 6:**
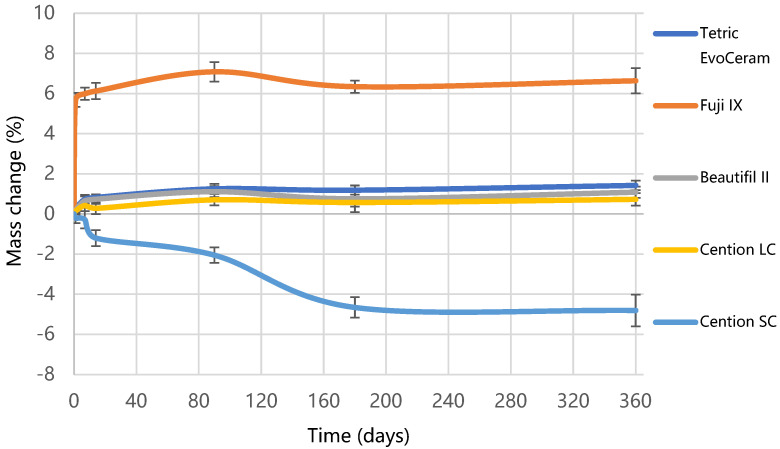
Mass change over one year of water immersion for tested materials. Error bars denote standard deviations.

**Figure 7:**
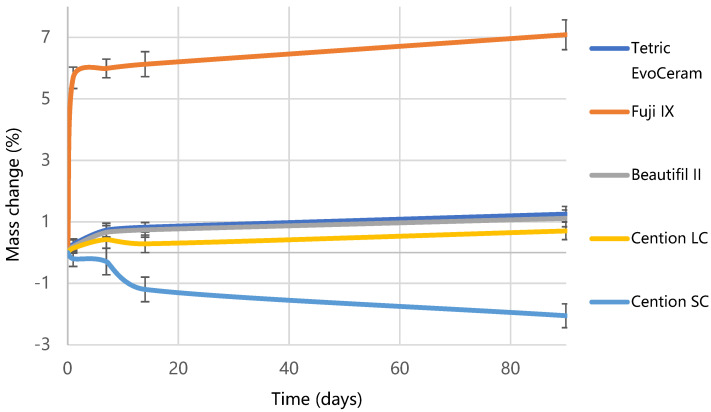
Mass changes over three months of water immersion. Error bars denote standard deviations.

Figure 5 shows the results of water sorption and solubility. Fuji IX exhibited the highest water sorption (127.6 µg/mm^3^), followed by Cention SC (73.6 µg/mm^3^), Cention LC (40.5 µg/mm^3^), while Tetric EvoCeram (31.6 µg/mm^3^) and Beautifil II (30.4 µg/mm^3^) had the lowest sorption (*p* < 0.001). The highest solubility was demonstrated by Cention SC (193.9 µg/mm^3^), which was significantly reduced (*p* < 0.001) by photo-polymerization in Cention LC (21.9 µg/mm^3^). Full water saturation was achieved after 90 days for Tetric EvoCeram and Cention LC, and after 180 days for Cention SC. After 365 days, a plateau of mass change was not reached for Beautifil II and Fuji IX, as visible from Figure 6. The highest mass gain for Fuji IX was accomplished during the first day (Figure 7), while the weight of Cention SC continuously dropped after the seventh day and continued falling for six months. At the 3-month point, Cention SC was the material with the lowest mass (*p* = 0.001–0.082), indicating mass loss. At the same time, Fuji IX had the highest mass (*p* < 0.001), while light-cured groups (Tetric EvoCeram, Beautifil II, and Cention LC) behaved statistically similarly.

**Figure 8:**
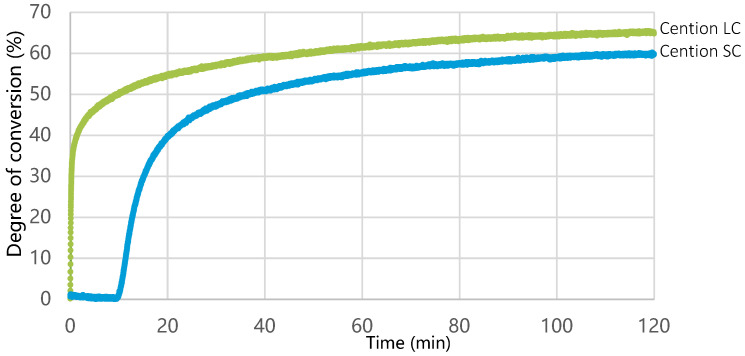
Degree of conversion as a function of time for Cention LC (green line) and Cention SC (blue line).

The increase in the degree of conversion for Cention LC started immediately after activation of the curing unit, while for Cention SC, it started 11 min after mixing (Figure 8). The degree of conversion after 120 min was significantly higher (*p* = 0.007) for Cention LC (65.0 ± 2.1%) than for Cention SC (59.7 ± 2.5%).

## 4. Discussion

This study examined the evolution of mechanical properties of ion-releasing materials over three months and after accelerated aging in ethanol, while water sorption and solubility were evaluated over one year. It was found that the flexural properties of the new bioactive composite Cention were higher than those of a high-viscosity glass-ionomer and lower than those of a conventional resin composite. When left to self-cure, this dual-cure bulk-fill material exhibited a slow increase in flexural strength and modulus as well as increased solubility. On the contrary, when light-cured, Cention showed slightly lower values than other light-cured materials in terms of mechanical properties and water sorption.

The distinct behavior of Cention in the self-cured and light-cured mode likely originated from different polymerization kinetics and resulting polymer networks. While the polymerization rate is the highest during light irradiation for the light-cured composites, redox polymerization in the self-curing modality has a delayed onset [38]. Ilie showed that initiation of polymerization of Cention N (hand-mixed) in a self-cure mode is lagging for 3.5 min after hand mixing, and that 11 min is needed to attain the same degree of conversion as in the light-cured mode [33]. However, our polymerization kinetics data on a capsulated Cention demonstrate the 11-min delay in initiating the polymerization of the self-cure mode, which prolongs the manufacturers’ claimed working time from 2 to 11 min and extends the claimed setting time of 6.5 min. In this study, the polymerization reaction was monitored over two hours, and the self-cured Cention never reached the same degree of conversion as when light-cured. This is in accordance with two recent studies that found a significantly lower degree of conversion of self-cured vs. light-cured for a majority of tested dual-cured resin composites [34,38].

A delay in the polymerization activation of Cention SC led to a quick drop in mass of the water sorption specimens, indicating high solubility. The solubility of Cention was likely related to the dissolution of the functional fillers in an aqueous environment. It is necessary to underline that the present study diverged from the ISO 4049 recommendations for self-cured polymer materials (Class 1) in preparing the specimens [39]. While the ISO recommends the 60 min setting time, we opted for a more clinically relevant 15 min setting. The apparent instability of the self-cured specimens was reflected in the initially low flexural strength (62 ± 13 MPa) and modulus (2.8 ± 0.5 GPa) of the 1-day specimens. This is in contrast to the previous study on a predecessor material Cention N that allowed the 60 min setting and found much higher 1-day values (~100–120 MPa flexural strength and ~4–5 GPa modulus) [33]. The observed discrepancies are evidently related to the study design and the compositional modifications that had to be made for adjustment to the trituration of a capsulated Cention. The mass loss of Cention SC continued at a 3-month time point, reaching the equilibrium only after 6 months. However, mechanical properties improved over time despite the solubility.

This behavior could be explained by the fact that dense and highly cross-linked polymer network yields higher strength and modulus of a resin-based composite [40,41]. Even though no long-term measurements of degree of conversion were made, we can hypothesize that the gradual development of polymer cross-linking could have contributed to a delayed increase in flexural strength and modulus in Cention SC. At the same time, self-curing enabled uniform polymerization throughout the entire specimen, which apparently led to a close distribution of flexural strength values and practically no aging-induced change in reliability for Cention SC. Unfortunately, initial flexural strength and modulus values fall below the values recommended by ISO 4049, so using this material without the additional light-curing is not advised. Light-curing of the surface could act as an umbrella, protecting the deeper layers from the detrimental influence of water. However, the flexural strength and modulus gradient could cause an uneven distribution of forces and material fracture during the service life of the restoration. Further investigations in this field are necessary.

In the present study, accelerated aging in ethanol diminished flexural strength only in the light-cured Cention specimens, but not the self-cured. This phenomenon could be attributed to a significantly higher refractive index of alkaline fillers in contrast to conventional inert glass fillers [33]. Due to large filler/resin refractive index discrepancy, higher opacity of Cention in comparison to other bulk-fill composites is noted. Consequently, low light transmission is found, leading to only 13% light penetrating the material at 2 mm depth and 3% at a 4 mm level [33]. Considering that the photoinitiators in Cention are dibenzoyl germanium derivative and an acyl phosphine oxide, photoactivation in the violet part of the spectrum around 409 nm is optimal. Unfortunately, violet wavelengths reach shorter depths than blue due to exponential light attenuation. Higher opacity and lower light transmission could have led to an inhomogeneous polymer network with a decreasing cross-linking density. Such heterogeneous networks consist of highly crosslinked microgel agglomerates surrounded by less cross-linked polymer [41,42]. Ethanol as an organic solvent quickly penetrates the parts of the polymer network with fewer chemical cross-links, separates physical (hydrogen) bonds, and causes plasticization of the resin [43,44]. This degradation of the polymer network was probably reflected in the reduction in strength and modulus for light-cured Cention in the 3 mth + eth group. Contrary, self-cured Cention presumably achieved more uniform cross-linking throughout the entire thickness of the specimen. However, the polymerization reaction for Cention SC occurred at a much slower pace, which led to statistical difference in flexural strength and modulus between 1-day and both 3 mth and 3 mth + eth groups.

Similar to Cention SC, an unexpected rise of flexural strength and modulus was observed for giomer Beautifil II after artificial aging in ethanol. In the present study, the specimens were subjected to ethanol exposure to provoke maximum plasticization of the organic matrix and thus give the worst possible outcome of flexural properties [43,45]. Beautifil II has the highest filler volume in this study and, therefore, less organic matrix that could be susceptible to plasticization [19]. Still, this is not a complete explanation for the unusual behavior demonstrated after ethanol exposure. To the authors’ knowledge, there are no studies that subjected Beautifil II to long-term water storage and ethanol after long-term water storage. However, its predecessor, Beautifil, was studied by Yap et al. [46]. They compared 30 days of water storage at 37 °C and 5000 thermal cycles varying between temperatures from 15 °C, 35 °C, and 45 °C. They found increased modulus and hardness after thermal cycling, explained by the post-cure polymerization due to heat exposure [46]. This explanation cannot be applied to the present study. Considering that the Beautifil II contains a traditional bis-GMA/TEGDMA matrix, we can only speculate that the ethanol-related increase can be associated with the unique S-PRG filler. However, the exact answer is still to be elucidated in future studies.

On the other hand, glass-ionomer Fuji IX was predictably the least reliable material in the study, with wide flexural strength data distribution, the highest water sorption, and generally lowest flexural properties. Such behavior is well described in the literature and can be attributed to high water sorption due to increased mobility of the sodium ion in the functional glass at room temperatures. Sodium is exchanged for hydrogen ions and causes hydrolytic instability and high solubility [14]. In addition to the hydrophilicity of glass-ionomers, internal porosities were identified as the origins of water accumulation, dissolution, and degradation of mechanical properties [14,15]. High water sorption of Fuji IX in the present study was thus expected and within the range of values described earlier [47,48,49]. Negative solubility for Fuji IX indicates incomplete water evaporation. Water was likely permanently bound during the cement’s maturation as this hydrophilic material uses water in the setting process [50]. Similar behavior, but to a smaller extent, was noted for the reference composite material Tetric EvoCeram. These negative solubility values were reported in previous studies for the same material [18,51]. The literature describes that the water remained bound by the hydrogen bonds to the -OH groups in the methacrylates of the resin matrix [52].

According to ISO 4049, the maximum allowed water sorption for a polymer-based restorative material is 40 µg/mm^3^ and 7.5 µg/mm^3^ for solubility [39]. Both self-cured materials in this study overstepped these limits, even though ISO 4049 does not apply to conventional glass-ionomers. Cention SC showed the highest solubility (194 ± 24 µg/mm^3^), while Fuji IX had the highest water sorption (127 ± 12 µg/mm^3^). The insufficient curing could again explain the solubility of Cention SC compared to Cention LC. Water absorbed in partially polymerized specimens could cause leaching out of the unpolymerized monomers and, to a smaller extent, dissolution of functional fillers, loss of mass, and higher solubility [50]. The most significant weight loss of Cention SC specimens occurred during the first six months after water immersion but continued up to one year, as illustrated in Figure 6 and Figure 7. Cention LC, on the other hand, behaves similarly to other light-cured materials. Thus, it is essential to reiterate that Cention should always be light-cured when placed in the oral cavity.

The hydrolytic deterioration of mechanical properties of polymer-based materials is significant [45,53], especially in ion releasing materials [54]. The time factor plays an important role in the diffusion of water or ethanol throughout the materials. The 24 h exposure to water proposed by ISO 4049 [39] seems insufficient to estimate the behavior of a material in a clinical setting [55,56]. Therefore, long-term studies such as the present one are necessary for evaluating ion-releasing materials. However, water sorption and solubility were not correlated to the decline of mechanical properties of all materials in this study. Previously, water sorption and flexural properties were related to filler type and amount, monomer composition, silanization and polymer crosslinking density [18,53,55]. The high filler ratio was the probable reason for the high long-term aging resistance of giomer Beautifil II, comparable or better than the inert reference material. On the other hand, high water sorption was likely the cause for the deterioration of the mechanical properties of glass-ionomer Fuji IX. New functional restorative material, alkasite Cention, showed a similar but slightly lower sorption and mechanical behavior pattern as an inert composite control, but only when light-cured. Considering the low ion-releasing ability of giomers [10,19], and poor mechanical properties of tested glass ionomer, it seems that alkasite Cention could be a viable ion-releasing alternative to conventional composite resins.

## 5. Conclusions

In conclusion, our results indicate that, when light-cured, Cention’s mechanical and water sorption properties are satisfactory and better than the mechanical properties of a glass-ionomer tested here. Leaving the Cention to self-cure will cause lower polymerization of the material, high solubility, and poor mechanical properties immediately after placement. Alkasite Cention should be used only in the light-cure mode.

## Figures and Tables

**Table 1:** The composition of the tested materials provided by the manufacturers.

Type	Product Name (Manufacturer)	Composition	Curing Mechanism
Alkasite	Cention (Ivoclar Vivadent)	Powder: inert barium alumino-boro-silicate glass, ytterbium fluoride, a calcium fluoro-alumino-silicate glass, and a reactive SiO_2_-CaO-CaF_2_-Na_2_O glass Liquid: UDMA, aromatic aliphatic UDMA, DCP, and PEG-400-DMA Initiator system: hydroperoxide, Ivocerin, and acyl phosphine oxide Filler content: 58–59 vol%	Dual-cure
Giomer	Beautifil II (Shofu Dental GmbH)	Fillers: s-PRG (aluminofluoro-borosilicate glass), Al_2_O_3_ Resin: bis-GMA, TEGDMA Filler content: 69 vol%	Light-cure
Glass-ionomer	Fuji IX GP Fast (GC Europe)	Powder: fluoro-alumino-silicate glass Liquid: Polybasic carboxylic acid (copolymer of acrylic and maleic acid), tartaric acid, water	Self-cure
Composite (control)	Tetric EvoCeram (Ivoclar Vivadent)	Fillers: Barium glass filler, ytterbium fluoride, mixed oxide, prepolymers Resin: bis-GMA, UDMA, bis-EMA Filler content: 53–55 vol%	Light-cure

Abbreviations: Bis-GMA—bisphenol-A-glycidyldimethacrylate; TEGDMA—triethylene glycol dimethacrylate; UDMA—urethane dimethacrylate; bis-EMA—ethoxylated bisphenol A-dimethacrylate; s-PRG—surface-modified pre-reacted glass-ionomer fillers.

## Data Availability

The datasets generated during and/or analyzed during the current study are available from the corresponding author on reasonable request.
